# Multiple polypoid lesions in the upper airways

**DOI:** 10.36416/1806-3756/e20260288

**Published:** 2026-08-13

**Authors:** Edson Marchiori, Bruno Hochhegger, Gláucia Zanetti

**Affiliations:** 1. Universidade Federal do Rio de Janeiro, Rio de Janeiro (RJ) Brasil.; 2. University of Florida, Gainesville (FL) USA.

**Figure 1:**
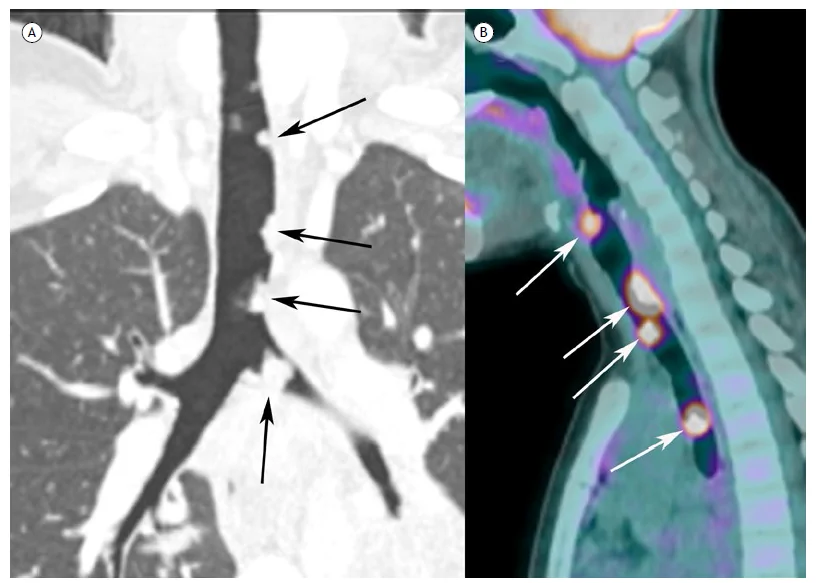
In A, Coronal CT of the thorax showing multiple nodular lesions in the trachea and bronchial walls (arrows). In B, 3D sagittal reconstruction of an 18F-FDG PET/CT image showing lesions in the trachea and bronchi with high glycolytic activity (arrows).

A 29-year-old woman, born by vaginal delivery, with a history of laryngeal papillomatosis since the age of 2, underwent multiple resections due to respiratory obstruction. She recently developed respiratory obstruction and pneumonia. Chest CT demonstrated multiple nodular lesions in the trachea and bronchial walls (Figure 1A). The patient underwent direct laryngoscopy and flexible bronchoscopy, which revealed papillomatous lesions of the vocal cords, trachea, and bronchi. ¹⁸F-FDG PET/CT demonstrated high glycolytic activity of the lesions in the trachea and bronchi (Figure 1B). Biopsy of the tracheal lesion was diagnostic of laryngotracheobronchial papillomatosis (LTBP).

LTBP is a benign condition caused by HPV. Although it is usually limited to the larynx, it can occasionally become aggressive and result in persistent and recurrent involvement of the nasopharynx, larynx, and tracheobronchial tree. Spread to the pulmonary parenchyma is rare. Infection most commonly occurs at birth, related to passage through the birth canal in infected mothers. In adults, HPV transmission occurs through oral sex. The disease course is unpredictable, ranging from spontaneous remission to pulmonary dissemination, requiring multiple surgical interventions. 

The definitive diagnosis of LTBP is made through bronchoscopy with biopsy, although it may be suggested by CT findings, with the identification of polypoid lesions in the airways associated with pulmonary nodules, which are frequently cavitated. The main differential diagnosis is hematogenous metastases. Other findings related to airway obstruction and/or associated infections include atelectasis, consolidations, air trapping, post-obstructive infection, or bronchiectasis.^1^ ^-^ ^3^

LTBP, in most cases, presents no diagnostic difficulty, since the pulmonary manifestation is typically preceded by a rich clinical history and an already established diagnosis of laryngeal papillomatosis, generally from childhood. The most feared complication is malignant transformation to squamous cell carcinoma. Imaging studies provide little information regarding malignant transformation in the absence of features of direct invasion or distant dissemination. Nodular papillomas and carcinomas are indistinguishable by chest X-ray or CT. ¹⁸F-FDG PET/CT is also of limited value in the differential diagnosis. The main evidence of malignant transformation is the abnormal growth of a nodule or mass. Recurrence of papillomas is common, regardless of the treatment used. In practice, these patients undergo frequent bronchoscopic surveillance, as recurrent resections are generally required.^1^ ^-^ ^3^
